# Snowshoe hare virus: discovery, distribution, vector and host associations, and medical significance

**DOI:** 10.1093/jme/tjad128

**Published:** 2023-10-20

**Authors:** Edward D Walker, Thomas M Yuill

**Affiliations:** Department of Microbiology and Molecular Genetics, Michigan State University, East Lansing, MI, USA; Department of Pathobiological Sciences, University of Wisconsin-Madison, Madison, WIUSA

**Keywords:** snowshoe hare virus, California serogroup, *Orthobunyavirus*, hosts, vectors, disease

## Abstract

Snowshoe hare virus (SSHV), within the California serogroup of the genus *Orthobunyavirus*, family *Peribunyaviridae*, was first isolated from a snowshoe hare (*Lepus americanus*) in Montana, United States, in 1959. The virus, closely related to LaCrosse virus (LACV) and Chatanga virus (CHATV), occurs across Canada and the northern latitudes of the United States, primarily in the northern tier of states bordering Canada. Reports of SSHV in northern Europe and Asia are probably the closely related to CHATV, or the less closely related Tahyna virus. Vertebrate associations include snowshoe hares and ground squirrels, demonstrated by field isolation of virus from wild-caught animals, seroconversion of snowshoe hares, seroconversion of sentinel rabbits, isolation of virus from sentinel rabbits, and experimental infections demonstrating viremia. Isolations of virus from field populations of mosquitoes include primarily univoltine and boreal mosquitoes of the genus *Aedes*, *Culiseta impatiens* and *Culiseta inornata*; and, rarely, certain multivoltine floodwater *Aedes* species. Experimental transmission studies in mosquitoes show infection in and transmission by boreal *Aedes* and *Culiseta inornata*. Isolation of SSHV from larval *Aedes* on three occasions, and experimentation in *Culiseta inornata*, reveal transovarial transmission of the virus in mosquitoes. Serosurveys reveal exposure to SSHV in human and domestic animals, with rates of seropositivity commonly high in some settings in Alaska and Canada, but disease in humans or horses has rarely been reported, only in Canada.

## Discovery

Snowshoe hare virus (SSHV) was first isolated from the blood of an emaciated snowshoe hare (*Lepus americanus* Erxleben), heavily parasitized with *Dermacentor andersoni* Stiles ticks, in the Bitterroot Valley of western Montana in 1959 by Willy Burgdorfer and colleagues (Burgdorfer et al. 1961, Calisher 1983, Grimstad 1988). It was later isolated from vertebrates and mosquitoes in the following decade, eventually recognized as a virus distinct from CEV and LACV (Sather and Hammon 1967) (Table 1). The discovery of SSHV was serendipitous; Burgdorfer and colleagues were conducting research on Colorado Tick Fever virus while working at the Rocky Mountain National Laboratories (Burgdorfer et al. 1961, Padgett et al. 2022). SSHV represents the fourth of the California (CAL) serogroup viruses to have been discovered in North America, since California encephalitis virus (CEV) was isolated from *Aedes melanimon* Dyar in 1943 in California, Trivittatus virus (TVTV) from *Aedes trivittatus* in 1948 in North Dakota, and San Angelo virus from *Anopheles punctipennis* (Say) in 1958 in Texas (LeDuc 1979, Calisher 1983, Calisher 1996, Grimstad 1988,). During the same period of time, Melao virus was isolated from *Aedes scapularis* (Rondani) in Trinidad, Tahyna virus from *Aedes caspius* (Pallus) in Czechoslovakia in 1958, and Lumbo virus from *Aedes pembaensis* Theobald in Mozambique in 1959 (LeDuc 1979). However, SSHV is the first of the CAL serogroup viruses to have been discovered from a vertebrate versus mosquito source (LeDuc 1979). The isolated agent was lethal upon infection to suckling mice but not to jackrabbits nor cottontail rabbits; the latter hosts oddly did not form viremia (Burgdorfer et al. 1961). At least in one experiment, the agent was transmissible via infected mosquitoes (*Aedes aegypti* [L.]) to suckling mice (Burgdorfer et al. 1961). McKiel et al. (1966) obtained 5 isolates of SSHV from sentinel domestic rabbits (*Oryctolagus cuniculus* [L.]) in Richmond, Ontario, in the summers of 1962–1963, the first isolation of any California serogroup virus in Canada and the second time SSHV was isolated from vertebrates in nature, but inadvertently reported by McLean et al. (1974) as from snowshoe hares. Subsequently, SSHV was isolated from a juvenile snowshoe hare in 1968 in Alberta (Hoff et al. 1971), and from sera (primarily) of 21 of the 162 snowshoe hares sampled by live trapping or shooting in east-central Alaska from 1969 to 1971 (Ritter and Feltz 1974). The latter study also reported isolation of SSHV from liver and spleen of a collared lemming (*Dicrostonyx groenlandicus* [Traill]) (reported as the varying lemming, *Dicrostonyx rubricatus* [Richardson]) and from brain of northern red-backed vole, *Clethrionomys rutilis* (Pallas). The latter event was a mixed infection of SSHV and Northway virus (a Bunyamwera virus). SSHV was not isolated from sentinel rabbits in Alaska (Ritter and Feltz 1974), as was done in Ontario (McKiel et al. 1966).

**Table 1.** Historical discovery of snowshoe hare virus (SSHV) by year of isolation and characterization, 1959–1971

Year	Isolation event	Location	Reference
1959	Isolation from a snowshoe hare	Montana, USA	Burgdorfer et al. (1961)
1962–1963	Isolation from *Aedes fitchii* and *Culiseta impatiens*	Montana, USA	Newhouse et al. (1971)
1962–1963	Isolation from sentinel, domestic rabbits	Ontario, Canada	McKiel et al. (1966)
1965	Isolation from *Culiseta inornata*	Alberta, Canada	Morgante and Shemanchuk (1967)
1965	Isolation from *Aedes communis* and *Aedes stimulans*	Alberta, Canada	Iversen et al. (1969)
1965	Isolation from *Aedes cinereus*	New York State, USA	Whitney et al. (1969)
1965	Isolation from *Aedes communis*	Wisconsin, USA	Sudia et al. (1971)
1966	Isolation from *Aedes vexans*	Wisconsin, USA	Sudia et al. (1971)
1968	Isolation from *Aedes communis*	Alberta, Canada	Hoff et al. (1971)
1968	Isolation from *Aedes canadensis*	Massachusetts, USA	Sudia et al. (1971)
1968	Isolation from a snowshoe hare	Alberta, Canada	Hoff et al. (1971)
1969–1971	Isolation from snowshoe hares, collared lemming, northern red-backed vole	Alaska, USA	Ritter and Feltz (1974)
1970–1971	Isolation from *Aedes excrucians Aedes punctor*, *Aedes communis Aedes cinereus*, *Aedes fitchii*, *Aedes hexodontus*, *Aedes intrudens*, unidentified *Aedes* spp., *Simulium* spp.	Alaska, USA	Sommerman (1977)

The temporal sequence of the original isolation of SSHV from mosquitoes is asynchronous with the temporal sequence of publication of these findings (Table 2). The first isolations of SSHV from mosquitoes came from the period 1962–1963 in valleys of the Bitterroot mountains of Montana, United States, near where the first infected snowshoe hare was trapped (Burgdorfer et al. 1961), but the results were published much later (Newhouse et al. 1971). Of 19,321 mosquitoes of 14 species in 869 pools, five isolates were obtained from 276 pools of 7,592 *Aedes fitchii* (Felt and Young) (MFIR, 1:1518), and 1 isolate was obtained from 188 pools of 4,763 *Culiseta impatiens* (Walker) (MFIR, 1:4763). The latter isolate was obtained from a pool of three blood-fed individuals aspirated from a trap baited with a domestic goat. Subsequently in 1965, SSHV was isolated from mosquitoes in four different locations (but published earlier than Newhouse et al. 1971), as follows. First, it was isolated from two pools of *Culiseta inornata* Williston sampled near Brooks and Hays-Vauxhall, southern Alberta, Canada (Morgante and Shemanchuk 1967). These mosquitoes were collected while resting in a rodent burrow, and were blood-fed, but the source of the blood was not determined. Accordingly, whether the mosquitoes themselves were infected or the blood in them was infected with SSHV is not known. Second, SSHV was isolated from three pools of *Aedes communis* (DeGeer) group and one pool of *Aedes stimulans* (Walker) group mosquitoes, near Rochester, Alberta, Canada (Hoff et al. 1969, Iversen et al. 1969). Third, SSHV was isolated from a pool of *Aedes cinereus* Wiedemann in St. Lawrence County, New York State (Whitney et al. 1969, Grayson et al. 1983). Fourth, SSHV was isolated from *Aedes communis* sampled in Wisconsin (Sudia et al. 1971). Thereafter, SSHV was isolated from *Aedes vexans* in Wisconsin in 1966 (Sudia et al. 1971); *Aedes communis* in Alberta in 1968 (Hoff et al. 1971); from *Aedes canadensis* (Theobald) in Massachusetts in 1968 (Sudia et al. 1971, Walker et al. 1993); and 48 times from pooled mosquitoes of several species (16 *Aedes excrucians* [Walker], 14 *Aedes punctor* [Kirby], 4 *Aedes communis*, 3 *Aedes cinereus*, 1 *Aedes fitchii*, 1 *Aedes hexodontus* [Dyar], 1 *Aedes intrudens* Dyar; 7 from unidentified *Aedes* spp.); and once from a pool of *Simulium* spp. (Diptera: Simuliidae), all in east-central Alaska in 1970–1971 (Sommerman 1977). A report of a single CEV isolation from a mixed pool of *Ae. canadensis* and *Ae. vexans* in southern British Columbia in 1969 (McLean 1970, Iversen et al. 1973) may have been SSHV. Artsob (1983) summarized isolations of SSHV from mosquitoes in Canada since the original isolations described above. Studies by McLean et al. in the Yukon Territory and the Northwest Territories of Canada represent one of the longest series of multi-year analyses of SSHV in mosquitoes in a particular region (summarized by McLean 1983), whose results from 1972 to 1982 revealed isolation of SSHV from mosquitoes in 8 of those 11 years. Interestingly, Wagner et al. (1975) report 4 isolates of SSHV from *Aedes hexodontus-punctor* from the Meliadine River region of the Nunavut Territory, a treeless tundra region well north of the distribution of snowshoe hares in Canada.

**Table 2.** Isolations of snowshoe hare virus from mosquitoes during the first decade of its discovery. Adapted from LeDuc (1979), Turell and Calisher (1983), Grimstad (1988), Evans et al. (2019) and from cited references

Species	Year	Location	No. of isolates	Reference
*Aedes fitchii*	1963	Bitter Root Mountains, Montana USA	5	Newhouse et al. (1971)
*Culiseta impatiens*	1963	Bitter Root Mountains, Montana USA	1	Newhouse et al. (1971)
*Culiseta inornata*	1965	southern Alberta, Canada	2	Morgante and Shemanchuk (1967)
*Aedes communis* ^*^	1965	Rochester, Alberta, Canada	3	Iversen et al. (1969)
*Aedes stimulans* ^*^	1965	Rochester, Alberta, Canada	1	Iversen et al. (1969)
*Aedes cinereus*	1965	St. Lawrence County, New York	1	Whitney et al. (1969)
*Aedes communis*	1965	Wisconsin	1	Sudia et al. (1971)
*Aedes vexans*	1966	Wisconsin	1	Sudia et al. (1971)
*Aedes communis* ^*^	1968	Alberta, Canada	1	Hoff et al. (1971)
*Aedes canadensis*	1968	Massachusetts	1	Sudia et al. (1971)
*Aedes spp.*	1969	Penticton, British Columbia, Canada	1	McLean (1970)
*Aedes excrucians*	1970–1971	east-central Alaska	16	Sommerman (1977)
*Aedes punctor*	1970–1971	east-central Alaska	14	Sommerman (1977)
*Aedes communis*	1970–1971	east-central Alaska	4	Sommerman (1977)
*Aedes cinereus*	1970–1971	east-central Alaska	3	Sommerman (1977)
*Aedes fitchii*	1970–1971	east-central Alaska	1	Sommerman (1977)
*Aedes hexodontus*	1970–1971	east-central Alaska	1	Sommerman (1977)
*Aedes intrudens*	1970–1971	east-central Alaska	1	Sommerman (1977)
*Aedes* spp.	1970–1971	east-central Alaska	7	Sommerman (1977)
*Simulium* spp.	1970–1971	east-central Alaska	1	Sommerman (1977)

^*^Reported as species group or species complex.

Isolations of SSHV from ticks (*Haemaphysalis leporispalustris* Packard, *D. andersoni*) removed from mammalian hosts were attributed to acquisition of virus from infected hosts; infections waned within 48 hours, leading to the conclusion that ticks do not become infected and are not vectors (Newhouse et al. 1963, Turell and LeDuc 1983). None of the 130 nymphal and adult *D. andersoni* taken from the snowshoe hare from which Burgdorfer et al. (1961) isolated SSHV originally were infected with the virus. Those ticks were held alive for 2 months after removal from the snowshoe hare to allow for time for blood digestion before virus isolation was attempted.

The above findings on vertebrates and mosquitoes, aside from demonstrating a new virus, established the association of SSHV with snowshoe hares (thus, the name of the virus) and mosquitoes, and the quite widespread geographic distribution of the virus. It is important to note that SSHV as an entity distinct from CEV was recognized gradually as other viruses such as LaCrosse (LAC) virus were discovered and as immunological reagents and procedures became available. Sather and Hammon (1967) in their comprehensive study of antigenic variation of the CEVs available at that time determined definitively that SSHV, while antigenically broadly reactive, was sufficiently distinct to be classified as one of the 8 prototype viruses of the CEV group, including differentiation from LACV to which it is closely related. Prior to this seminal work, it was not clear if SSHV was simply a northern equivalent of CEV or identical to LACV. However, the classification of SSHV is upheld well with serum dilution, neutralization tests (Lindsey et al. 1976). The findings reviewed above also show that infection with SSHV is not limited to snowshoe hares but includes other species of small mammals, particularly ground squirrels; and that a wide range of mosquito species in the genera *Aedes* and *Culiseta* are infected in nature. Despite this legacy of illuminating research, there are several important gaps in knowledge of the SSHV transmission system and much is inferred from indirect information, or concluded without a strong foundation of data.

## Distribution

**Fig. 1.**
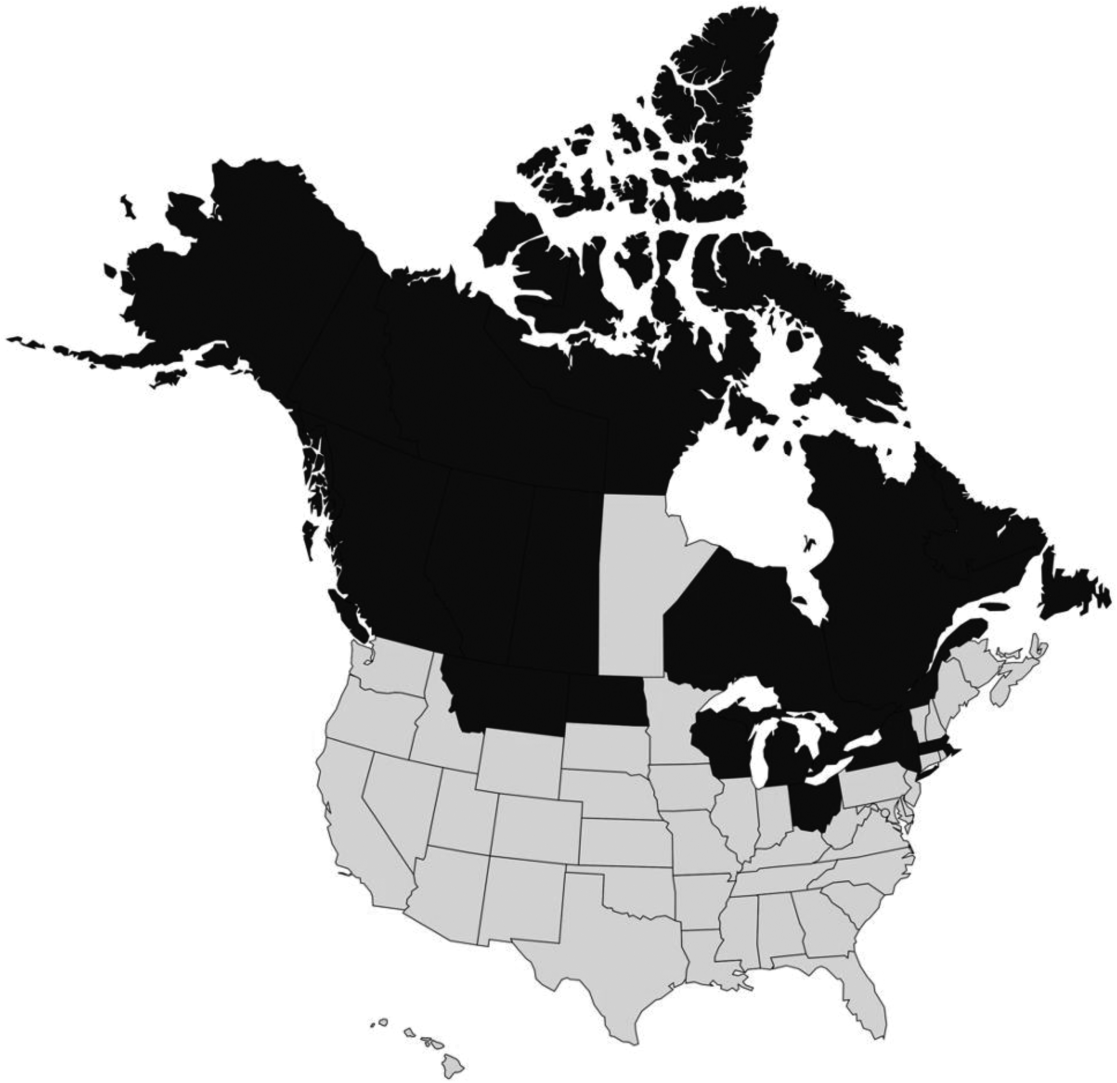
Provinces of Canada and states of the USA (dark color) where snowshoe hare virus has been isolated from vertebrates or mosquitoes (see Tables 1–2).

A map of the provinces and territories of Canada, and states of the United States where SSHV has been isolated from vertebrates or insect vectors is shown in Fig. 1. Evans and Peterson (2019) and Evans et al. (2019) provide maps of the distribution of SSHV and other CAL serogroup viruses. When considering both isolation of virus from vertebrates or mosquitoes, and serosurveys using SSHV as the antigen, SSHV is broadly distributed in boreal regions and northern latitudes of North America, occurring in Canada from the Yukon Territory east through the present Northwest Terrorities and Nunavut Territory to the maritime provinces and Newfoundland of Canada; and in Alaska, Montana, Wyoming, North Dakota, Wisconsin, Ohio, New York State, and Massachusetts (Sudia et al. 1971, Wagner et al. 1975, Artsob et al. 1982, Berry et al. 1983, Calisher 1983, Grayson et al. 1983, McLean 1983, Turell and LeDuc 1983, Mokry et al. 1984, Grimstad et al. 1988, Walker et al. 1993, Anderson et al. 2015, Evans and Peterson 2019, and Evans et al. 2019). McLean et al. (1974) argued that SSHV is a virus of northern latitudes, particularly north of 45 °C N, and that it is the predominant mosquito-borne virus in North America north of latitudes 53–54 °C N. The rarity or lack of isolations of SSHV from mosquitoes in states below the above-named northern tier in the United States, such as Iowa (no isolates from 1966 to 1980 despite numerous TVT virus isolates, from analysis of 498,268 mosquitoes; Rowley et al. 1983) and Ohio (only two isolates over 18 years from analysis of 1,391,118 mosquitoes; both from *Aedes trivittatus* Coquillett; Berry et al. 1983) suggest that SSHV is a virus restricted to northern latitudes. Although snowshoe hares exist in Connecticut and surveys for mosquito-borne viruses have been done in that state since at least 1966, SSHV has yet to be isolated from mosquitoes sampled there (although Jamestown Canyon virus is present, as large-scale surveys for mosquito-borne viruses reveal; Sprance et al. 1978, Andreadis et al. 2008). Despite serologic evidence for SSHV infection in mammals in California (Campbell et al. 1990), the virus has not been isolated from mosquitoes in that state (Sudia et al. 1971, Campbell et al. 1991) nor was a local strain of SSHV available for use as antigen in the serosurvey carried out by Campbell et al. (1990). Thus, after decades of arbovirologic surveillance and research in California including in alpine regions with success at isolation of Jamestown Canyon virus (Campbell et al. 1991), we must exclude California from endemicity for SSHV including its alpine regions. The same can be said for Oregon, where serologic data suggest exposure of wild mammals to SSHV but where the virus has not been isolated from mosquitoes or vertebrates (Eldridge et al. 1987). Although Calisher (1983) reports SSHV from Minnesota, the primary literature reporting it is not available. Sudia et al. (1971) do not report SSHV from Minnesota in their comprehensive review, when LACV had been isolated numerous times from *Aedes triseriatus* around a focus on human disease there. There is no record of isolation of SSHV from Manitoba, a province that borders on the provinces of Ontario and Saskatchewan and the Nunavut Territory, from where SSHV has been isolated from both mosquitoes and mammals (Artsob 1983). Sudia et al. (1971) report an isolation of SSHV from *Aedes communis* in 1965 and from *Aedes vexans* in 1966 from Wisconsin, citing the source of information as unpublished data from R.O. Anslow, R.P Hanson, W.H. Thompson, and G.R. DeFoliart. Similarly, Issel et al. (1972) used the prototype strain of SSHV in experimental infections with deer, but noted a personal communication from Ralph P. Anslow that SSHV had been isolated in Wisconsin. These two instances appear to be the only reports of SSHV in that state where there has been intense focus on the California serogroup of viruses due to the presence of LACV there, named after the city of LaCrosse, Wisconsin.

Records of SSHV in northern Asia and northern Europe (Traavik et al. 1978, Mitchell et al. 1993, Lundström 1999, Putkuri et al. 2014, 2016) are probably Chatanga virus or Tanhyna virus, mosquito-borne California serogroup viruses closely related to SSHV, and causative agents of human infection and disease (Putkuri et al. 2016, Hughes et al. 2017, Evans and Peterson 2019, and Evans et al. 2021). A serosurvey of small mammals including shrews (genus *Sorex*) in western Siberia documented high seropositivity rates to SSHV (80.4% of the 184 individuals); however, only a single putative isolate of SSHV was obtained in the same study from a single *Aedes euedes* Howard, Dyar, and Knab mosquito. This isolation, which occurred at a different location than where the mammals were sampled and also resulted in isolation of a Bunyamwera group virus, prompted the investigators to note: “it is uncertain whether the test we used is specific for antibody to SSH …. It is clear from the results, however, that SSH virus *or a closely related virus* is present in the area” (Mitchell et al. 1993) [Authors’ italics]. Lacking further evidence, SSHV should be considered primarily a North American virus. Snyman et al. (2023) predicted that as global warming proceeds with greater intensity in the Nearctic region, SSHV will retain its current range in its southerly limit yet expand its range northward as habitat for vectors and vertebrate hosts becomes available. By contrast, Sultaire et al. (2016) documented a northward contracting southern range boundary of snowshoe hares and related it to a combination of habitat changes and loss of snow cover due to climate change. Further, Rochlin et al. (2016) observed that the range of species of mosquitoes with more northern distributions, such as those boreal and univoltine species associated with SSHV, also have southern distribution limits contracting northward. If such proves to be the case, the geographic distribution of SSHV could also shift northward as its southern range loses both a key vertebrate host and primary vector species from more southerly areas.

## Classification

The phylogenetic placement of SSHV and its taxonomic associations with other viruses was based initially upon relationships established by antigen/antibody interactions in hemagglutination-inhibition, complement-fixation, and neutralization tests (SatherSather and Hammond 1967, Lindsey et al. 1976, Calisher 1983, 1996, and Evans et al. 2021), later by comparison of single-strand conformational polymorphisms of the small RNA segment (Black et al. 1995), and most recently by analyses of nucleotide sequences for the small, medium, and large RNA segments as well as the predicted amino acid sequences from genes of these segments (Hughes et al. 2017). Collectively, these approaches show that SSHV is a virus distinct from the prototype CEV but which belongs to the CEV complex, within the California serogroup of viruses of the genus *Orthobunyavirus*, within the family Peribunyaviridae, order Bunyavirales (Calisher 1983, 1996, Hughes et al. 2017, 2020, and Evans et al. 2021). Serologic analyses originally indicated that SSHV is a variety of LACV, which in turn is a subtype of the CEV type strain (Lindsey et al. 1976, Calisher 1983, 1996). Bayesian Maximum Clade Credibility time tree analyses separately done on the nucleotide sequences of open reading frames of the small, medium, and large segments of the RNA genome reveal that the closest related virus to SSHV is Chatanga virus, and next closest is La Crosse virus, but SSHV is distinct from both as revealed by phylogenetic branching and percent similarity of nucleotide sequences or predicted amino acids (Hughes et al. 2017) (Table 3).

**Table 3.** Percent similarity of nucleotide sequences and amino acid sequences of the open reading frames of the small, medium, and large RNA genome segments of snowshoe hare virus compared with Chatanga, La Crosse, California encephalitis, and Jamestown Canyon viruses. Adapted from Hughes et al. (2017)

Virus segment	Variable		California serogroup virus^*^		
		CHAT	LAC	CE	JC
Small segment	Nucleotide sequence	89.9	89.1	82.1	78.4
	Amino acid sequence	93	90.9	89.1	81.7
Medium segment	Nucleotide sequence	80.7	79.4	73.2	69.3
	Amino acid sequence	65.8	64	57.6	53.1
Large segment	Nucleotide sequence	81.9	82	78.4	74.3
	Amino acid sequence	74.9	75.1	69	63.3

^*^CHAT, Chatanga; LAC, LaCrosse; CE, California encephalitis; JC, Jamestown Canyon.

## Virus Relationship to Vectors

Incriminating arthropods as vectors of blood-borne pathogens including viruses requires satisfying certain stringent criteria (formulated in Barnett [1962] and summarized in Merritt et al. [2010]): (1) experimental demonstration that the postulated vector acquires the virus from an infected vertebrate host, and becomes infected with the pathogen thereafter, (2) the postulated vector must have close physical associations with infected hosts, and feed upon the blood of those hosts, (3) the postulated vectors must be collected in endemic settings and found repeatedly to be infected with the pathogen in endemic settings where vertebrates are also found infected, and (4) the postulated vector must transmit the pathogen by bite or some other means (defecation, direct contact) under experimental conditions. Only some of these criteria have been met for SSHV in the case of mosquitoes.

Addressing criterion #3 first, snowshoe hare virus has been isolated from at least 17 species or species groups of mosquitoes, as well as from a *Simulium* spp. black fly and two species of ticks (*Dermacentor andersoni* and *Haemaphysalis leporispalustris*) (LeDuc 1979, Belloncik et al. 1983, McLean 1983, Turell and LeDuc 1983, Mokry et al. 1984, Grimstad 1988, Anderson et al. 2015, and Evans and Peterson 2019). Isolations have come repeatedly and primarily from two different groups of mosquitoes with quite different life histories. First are the univoltine and boreal *Aedes* species (including representatives of both the “dark-legged” univoltine species, referring to those without bands of white scales on the tarsal segments) of the *Aedes communis* group including the *Aedes punctor* subgroup; and the “stripe-legged” species such as *Aedes stimulans* and related species in a species complex sometimes called the *Aedes stimulans* group, as well as *Aedes canadensis* which does not belong to this group of species. However, isolations outside of these groups include *Aedes cinereus* (Whitney et al. 1969). Second, is *Culiseta inornata* Williston and *Culiseta impatiens* in the northwestern geographic range of its distribution (Turell and LeDuc 1983). That Turell and LeDuc (1983) designated *Culiseta inornata* as the principal vector of SSHV seems unwarranted, given the wide range of *Aedes* species from which the virus has been repeatedly recovered, the original isolation of SSHV not from *Culiseta nornate* but rather *Culiseta impatiens* (Newhouse et al. 1971), the fact that the first isolation of SSHV from *Culiseta nornate* was from blood-fed individuals, and the lack of reported isolates from *Culiseta inornata* in the eastern part of that species’ range. From the history of isolation of SSHV from mosquitoes, it would appear that many different species could function as natural vectors, or at least they ingest the virus from vertebrate blood in nature and harbor viral infection thereafter.

SSHV has been isolated occasionally and uncommonly from multivoltine, temperate, floodwater species such as *Aedes vexans* in Wisconsin (Sudia et al. 1971) and North Dakota (Anderson et al. 2015), and *Aedes trivittatus* in Ohio (Berry et al. 1983). However, Artsob (1983) noted that no viruses of the CAL serogroup were isolated from 46,413 *Aedes vexans* sampled in Ontario from 1976 to 1978, suggesting that SSHV does not circulate in populations of that species. Collectively, surveys of SSHV associations with mosquitoes support a model of high-latitude species and little role for multivoltine *Aedes* of lower latitudes. This association sets SSHV apart from the other CAL serogroup viruses such as Keystone virus and TVT virus, for which multivoltine floodwater *Aedes* species are important and for which rabbits serve as vertebrate hosts (LeDuc 1979, 1987). By contrast, SSHV and JCV share associations with univoltine, boreal *Aedes* species and *Culiseta inornata* (Turell and LeDuc 1983, Andreadis et al. 2008, and Farquhar et al. 2022), but to the best of our knowledge these two viruses have never been isolated from the same pool of mosquitoes.

Addressing criterion #4 and criterion #1, Burgdorfer et al. (1961) conducted experiments using their initial isolation of SSHV from a snowshoe hare with suckling mice and *Aedes aegypti* and *Culex tarsalis* mosquitoes, and demonstrated in the case of the former species transmission from infected suckling mice to mosquitoes and back to uninfected suckling mice, but were unable to repeat these observations with a second experiment. *Culex tarsalis* was an incompetent vector. McLean et al. (1977a) compared susceptibility to infection and transmission by *Aedes communis* and *Aedes aegypti* to a low passage strain of SSHV. Results showed that *Aedes communis* became infected at lower dosages and transmitted to suckling mice after 13 days or more when held at 13 or 23 °C, whereas *Aedes aegypti* became infected and transmitted only when fed a much higher viral dosage. Accordingly, these authors concluded that SSHV was adapted to boreal mosquito species compared to tropical species. McLean (1983) summarized findings of a series of experimental studies of infection and transmission of SSHV by *Culiseta inornata* and *Aedes communis* when temperature, incubation time, viral infectivity dose, and route of infection (intrathoracic inoculation or oral infection by feeding on infected mice) were varied (see McLean et al. 1975b, 1976, 1977a, and 1979). Mosquitoes of both species were found to become infected by both routes of virus exposure, resulting in evidence of infection in salivary glands, infection for hundreds of days when held at low temperatures (0 to 4 ^°^C), and transmission of virus to uninfected mice by bite, after which the suckling mice became infected. These findings support criteria #1, #3, and #4 for SSHV in relationship to *Culiseta inornata* and *Aedes communis*. Additionally, they suggest that SSHV could persist in overwintering populations of *Culiseta inornata* females. Heard et al. (1991) infected field-caught *Aedes provocans* (Walker) and *Aedes abserratus-punctor* with SSHV via membrane feeding, and after holding the mosquitoes at 19 ^°^C for > 12 days demonstrated transmission of virus to suckling mice by mosquito bite of 4/20 *Aedes provocans* and 2/20 *Aedes abserratus-punctor*. Hewlett et al. (1992) showed that LACV, two field strains of SSHV from northwestern Canada, and three high-passaged strains of SSHV were all transmissible by *Aedes triseriatus* by bite-to-suckling mice. Beaty et al. (1982) showed that LACV and SSHV reassortants varied in their transmissibility in *Aedes triseriatus*; they attributed this variation to reduced viral dissemination from the midgut in the presence of LAC viral reassortants containing the SSHV middle-sized segment of the SSHV genome.

Transovarial transmission was not studied in the above experiments, but McLean et al. (1975a, 1977b) separately isolated SSHV from *Aedes* mosquito larvae at endemic sites and indeed used an isolate in some of their adult mosquito infection experiments. SSHV was also isolated from adult *Aedes implicatus* Vockeroth raised from larvae collected in Saskatchewan province of Canada (McLintock et al. 1976) and from adult *Aedes communis* collected as larvae in Quebec province of Canada (Belloncik et al. 1982), suggesting natural, transovarial transmission. Schopen et al. (1991) showed experimentally that both *Aedes triseriatus* Say and *Culiseta inornata* transmitted SSHV transovarially after oral infection. Based on these findings collectively, *Culiseta inornata* could serve as vector and overwintering reservoir (inasmuch as adult females are in the overwintering stage), and as a maintenance species with efficient transovarial transmission of SSHV to progeny, while univoltine *Aedes* spp. such as *Aedes communis* could serve as vectors and overwintering reservoirs via transovarial and transstadial transmission. Newhouse et al. (1971) presented a graphical model portraying this system seasonally.

Although experimental infections have been done with snowshoe hares as well as species of rodents living in an endemic area of SSHV in Montana (Newhouse et al. 1971, Seymour et al. 1983), there have been no experiments involving experimentally or naturally infected snowshoe hares upon which mosquitoes were allowed to feed, followed by confirmation of infection of those mosquitoes, or transmission by infected mosquitoes to these hosts. Hoff et al. (1969) speculated that snowshoe hares may develop viremias sufficient to provide infectious blood meals for vector mosquitoes. However, viremia in snowshoe hares was lower and of shorter duration than for golden-mantled ground squirrel *Callospermophilus lateralis* Merriam, yellow pine chipmunk *Tamius amenous* Allen, or meadow vole *Microtus pennsylvanicus* Ord (Newhouse et al. 1971). In recognizing the problem of inferring that putative natural vertebrate hosts are infectious to mosquitoes in nature based upon laboratory transmission studies for JCV and SSHV, Heard et al. (1991) commented that “It is recognized that differences in mosquito infection rates can occur between vertebrate blood meals and artificial blood meals of equal titer. Thus, it is unclear whether the effective [infectious blood meal] titers employed in [our] trials overlapped the range of titers found in naturally infected wild vertebrates.” McLean et al. (1975a) observed “continued amplification of SSH virus in the Yukon in 1974 in the virtual absence of snowshoe hares [and] postulated that the ground squirrel (*Citellus undulatus*) was the main natural reservoir host in the summer of 1974” (Artsob 1983). This ground squirrel is now classified as the arctic ground squirrel, *Urocitellus parryii* Richardson (McLean 2018). But as with snowshoe hares, there are no experimental data on transmission of SSHV from arctic ground squirrels to mosquitoes. Many of the isolates of SSHV from snowshoe hares in east-central Alaska were obtained after the mosquito season had ended and cold weather commenced (Ritter and Feltz 1974), offsetting the timing of seasonal viremia with mosquito biting activity at least in part. Given these observations and findings, the essential role of snowshoe hares in SSHV transmission is unclear, despite the nomenclature of the virus.

Regarding criterion #2, blood meal analyses of putative vectors such as *Culiseta inornata* and *Aedes communis* are uncommon and not informative. Downe (1960) did not detect lagomorph blood meals in mosquitoes sampled in the Ottawa Valley near Ottawa, Ontario, where SSHV is likely endemic. Shemanchuk et al. (1963) did not include reagents for lagomorph blood meal identification in their studies of feeding habits of Alberta mosquitoes; *Culiseta inornata* fed upon large mammals in that study. Sommerman (1977) retained blood-fed mosquitoes for analysis in a sample from Alaska, but no host identification data were provided in that study. Anderson and Gallaway (1987) provided the most comprehensive analysis of blood host selection by *Culiseta inornata* in Canada where SSHV is endemic. They included reagents to detect lagomorphs, but there were no blood meals identified as snowshoe hares or any lagomorphs and all blood meals were from large-sized mammals. Consequently, there is a dearth of studies on host associations of mosquitoes in relationship to SSHV transmission, thus criterion #2 is not yet satisfied; rather, mosquito feeding on snowshoe hares appears not to have been demonstrated in SSHV endemic settings, (Hall 1984). Nonetheless, mosquitoes associated with transmission of other CAL serogroup viruses such as CEV, KEYV, and TVTV have regularly been demonstrated via blood meal analysis to feed upon wild rabbits (Pinger and Rowley 1975, Tempelis 1975, and LeDuc 1979).

Eldridge (1990) inferred evolutionary relationships between *Aedes* spp. mosquitoes (including *Ochlerotatus* and other subgenera) and California serogroup viruses by considering *Aedes* phylogeny and species groupings and the history of isolation of various viruses from different species of mosquitoes. Eldridge (1990) concluded that SSHV evolutionary associations with mosquitoes broadly encompassed both species of the *Aedes communis* group including the *Aedes punctor* subgroup, the *Aedes stimulans* group, and the *Aedes canadensis* group. He further noted that these associations were similar to those of Jamestown Canyon virus. Of interest is that these two viruses are rarely found in the same pools of mosquitoes, but there are examples of co-occurrence of JCv and SSHV in sympatric mosquito populations (Iversen et al. 1973), thus vertebrate host relationships must be a key variable to include in establishing such associations in nature.

## Serosurveys of Vertebrates

Much of what is known about associations of SSHV with vertebrates comes from results of serosurveys using SSHV as antigen in various serological tests (LeDuc 1979, Artsob 1983, Grimstad 1988). Serosurveys of snowshoe hare populations suggest variable but often high rates of exposure of snowshoe hares to this virus in Alaska, some provinces of Canada, and in Yellowstone National Park of Wyoming, USA (Newhouse et al. 1963, Hoff et al. 1969, Ritter and Feltz 1974, Artsob 1983, Rhyan et al. 2015). Newhouse et al. (1963) tested sera from snowshoe hares for neutralization antibodies to SSHV, and found the following to be positive: 5/94 (5.3%; Montana, USA), 8/9 (88.9%; upper peninsula of Michigan, USA), 3/6 (50%; Manitoulin Island, Ontario, Canada), 13/14 (92.9%; Kamloops, British Columbia, Canada), and 25/42 (59.5%; Richmond, Ontario, Canada). This study was the first one conducted on such a wide scale for what at the time was a new virus. Ritter and Fultz (1974) found 13/44 (29.5%) snowshoe hares with neutralizing antibodies to SSHV in eastern Alaska. McLean et al. (1975) sampled small mammals in the Yukon Territory from 1971 to 1974 and tested sera for neutralizing antibodies to SSHV; results showed that 430 of the 1,076 (40%) snowshoe hares, 266 of the 3,610 (7%) ground squirrels (*Citellus undulatus*, *viz*. *Urocitellus parryii*), and 9 of the 227 (4%) red squirrels (*Tamiasciurus hudsonicus* Erxleben) were positive. In Nova Scotia, 6%–14% of snowshoe hares had antibodies to SSHV, with variations among sampling sites (Embil et al. 1978). Follow-up surveys revealed continued seropositivity in snowshoe hare populations in that province (Embil et al. 1984). On Prince Edward Island, SSHV antibodies were found in wild and domestic animals with 15% of snowshoe hares positive by hemagglutination test and confirmed by neutralization test (McFarlane et al. 1981a), whilst in Alberta, 63% of snowshoe hares had neutralizing antibodies to SSHV (Zarnke and Yuill 1981). In southern Ontario, 25 of the 280 snowshoe hares were positive for SSHV antibodies (Artsob et al. 1982). In Newfoundland, virus neutralization seroprevalence rates for SSHV ranged to as high as 55%, and differed significantly amongst hares occupying discrete habitats separated by up to 35 km (Goff et al. 2012). Antibodies to SSHV have been found in other species of wild mammals, including moose (*Alces alces americana*) in Nova Scotia (McFarlane et al. 1981b), and mule deer (*0docoileus hemionus hemionus*), black-tailed deer (*O. hemionus columbianus*), and Roosevelt elk (*Cervus elaphus roosevelti*) in Oregon (Eldridge et al. 1987). Buhler et al. (2023) reported IgM antibodies to JCV and/or SSHV, as measured by a competitive ELISA procedure, in sera of caribou (*Rangifer tarandus* [L.]), Arctic fox (*Vulpes lagopus* [L.]), red fox (*Vulpes vulpes* [L.]), and polar bear (*Ursus maritimus* Phipps), and attributed rising seroprevalence from 1986 to 2017 to increasing temperatures in the arctic regions of Canada where the samples were acquired. Forty of 100 (40%) snowshoe hare sera were positive for antibodies to SSHV in studies conducted from 2009 to 2012 in the northern Greater Yellowstone area of Wyoming, USA. Of the six animals that were captured twice with at least one year between captures, four had developed antibodies to SSHV at the second capture (i.e., seroconverted), indicating active transmission of the virus (Rhyan et al. 2015). Snowshoe hares and a sentinel rabbit showed seroconversions to SSHV virus from May to August of 2010 and 2011 in Newfoundland, corresponding to the timing of the mosquito transmission season and when viral RNA was detected in simultaneously collected mosquitoes (Carson et al. 2017).

## Equine Infection

Antibodies to SSHV have been found frequently in domestic horses in Canada, indicating that they had been exposed to SSHV, presumably by mosquito bite, but did not develop clinical disease. For this reason, Artsob et al. (1978) proposed that horses were good indicators of the presence of the virus in southern Ontario. In Prince Edward Island province, 20% of equine animals were positive by hemagglutination test and confirmed by virus neutralization test (McFarlane et al. 1981a). In Nova Scotia, 72 of the 861 horses tested had neutralizing antibodies to SSHV (McFarlane et al. 1980). These SSHV infections in serologically positive horses were apparently asymptomatic. A survey of 115 paired equine serum and cerebrospinal fluid samples from horses with encephalomyelitis in southern Ontario were tested by hemagglutination-inhibition for SSHV antibodies, and by virus isolation. Fifty-one sera (44%) and 15 cerebrospinal fluids (13%) had antibodies to SSHV but no virus was isolated (Keane et al. 1988).

There have been two confirmed cases of equine encephalitis in Canada where SSHV was likely the causative agent. In July, 1983, a young stallion in Ontario exhibited acute encephalitis with mild fever, ataxia, head tilt, and circling behavior. Serology by hemagglutination-inhibition, complement-fixation, and neutralization tests revealed a 4-fold increase in antibody titer to SSHV. The horse recovered after 1 week with no sequelae (Lynch et al 1985). In June, 1987, a year-old filly in southern Saskatchewan developed neurological symptoms, and exhibited depression and muscle twitching upon admission. On the fourth day, she had spontaneous motor activity, dysphagia, and a single, short, generalized seizure. Her condition improved and she was discharged with complete recovery after 11 days. SSHV infection was confirmed serologically with virus neutralization tests on blood samples taken on days 2, 12, and 59 of illness (Heath and Bell 1988, Heath et al. 1989).

## Human Infection

There is serological evidence of human SSHV infection in several provinces in Canada spanning decades of investigation. In the area of Trois-Riviere, Quebec during 1974–1978, 6 of the 2,334 human sera were positive for antibodies to SSHV by the hemagglutination-inhibition test, three of which were confirmed by neutralization test (Belloncik et al. 1983). In southern Ontario, antibodies to SSHV were found in 23/406 human sera (Artsob et al. 1982). In a publication summarizing abstracts from four population-based studies conducted in Cree Territory, Quebec, between 2005 and 2009, seroprevalence to SSHV varied from 1% to 42% in different communities (Sampasa-Kanyinga et al. 2013). In New Brunswick, 3 of the 452 serum samples were positive for SSHV neutralizing antibodies (Mincer et al. 2021). None of these studies indicated that serologically positive individuals became ill as a result of their infections. Studies in east-central Alaska showed widespread exposure of an Aboriginal population of people to California serogroup viruses (Feltz et al. 1972), suspected to be related to SSHV exposure (Ritter and Feltz 1974).

Despite the close relationship of SSHV to LACV, and the significance of the latter as a causative agent of human infection and neurologic disease, there have been very few cases of illness in humans with documented infection with SSHV, and none reported from the United States. A total of 10 human cases of meningoencephalitis likely due to infection with SSHV were reported between 1978 and 1981 in Quebec, Ontario, and Nova Scotia, Canada (Fauvel et al. 1980, Artsob et al. 1981, Artsob 1983). Meier-Stephenson et al. (2007) reported a human case of meningoencephalitis in a 3-year-old boy from Nova Scotia. On admission, the case patient was pale, quiet, and febrile with a temperature of up to 40°C. On the second day in hospital, he experienced a tonic-clonic seizure following a period of decreased responsiveness. On the fourth hospital day his fever abated, he began to drink and eat, and became interested in play. Serology for antibodies to eastern equine, western equine, West Nile, St. Louis, Powassan, and dengue viruses were negative. An acute phase serum taken 3 days after the onset of illness was positive for IgM and IgG antibodies to SSHV using an antibody-capture enzyme-linked immunosorbent assay and immunofluorescence antibody testing. A convalescent-phase sample was obtained 25 days after illness; neutralization tests confirmed a greater than 4-fold rise in antibody titer in acute (1:320) and convalescent samples (1:5,120) for SSHV-specific antibodies. Two weeks after discharge, the case-patient’s parents reported that the child was completely well. Lau et al. (2017) described a case of meningoencephalitis due to SSHV infection in a 24-year-old Aboriginal male living in northern Manitoba. The case-patient exhibited one week of headache, nausea, and vomiting, altered mental status, a diffuse macular rash, recent exposure to mosquitoes, no history of travel outside Manitoba, and a fever of 39.1 ^°^C. Immunoglobulin M for JCV and SSHV were detected using IgM antibody-capture ELISA (MAC-ELISA). Sera collected at 8 and 28 days after onset of clinical symptoms were tested in parallel using plaque reduction neutralization (PRNT). The titer of SSHV-specific neutralizing antibody by PRNT was 4-fold greater than that of JCV; SSHV was identified as the etiological agent involved with this case.

Based upon the above findings and widespread seropositivity to both SSHV and JC virus (another California serogroup virus) in the Canadian human population and in the population of human diagnostic samples associated with meningoencephalitis (Makowski et al. 2009), Mincer et al. (2021) recommended differential diagnosis for undiagnosed febrile and neuroinvasive illness during the mosquito season to include CAL viruses as antigens, particularly when testing for common etiologies is negative or inconclusive.
